# Rheological, Mechanical and Morphological Characterization of Fillers in the Nautical Field: The Role of Dispersing Agents on Composite Materials

**DOI:** 10.3390/polym12061339

**Published:** 2020-06-12

**Authors:** Silvia Vita, Rico Ricotti, Andrea Dodero, Silvia Vicini, Per Borchardt, Emiliano Pinori, Maila Castellano

**Affiliations:** 1 Department of Chemistry and Industrial Chemistry, University of Genoa, Via Dodecaneso 31, 16146 Genova, Italy; silvia.vita@boero.it (S.V.); maila.castellano@unige.it (M.C.); 2 Boero Bartolomeo S.p.A., R&D “Riccardo Cavalleroni”, Strada Comunale Savonesa 9, PST—Blocco F, Rivalta Scrivia, 15057 Tortona, Italy; r.ricotti@boero.it; 3 Bioscience and Materials, RISE Research Institute of Sweden, Lindholmspiren 7 A, 41756 Göteborg, Sweden Borås, Sweden; per.borchardt@ri.se (P.B.); emiliano.pinori@ri.se (E.P.)

**Keywords:** nautical fillers, extender-matrix interactions, dispersing agents, mechanical properties, rheological properties, Payne effect, morphological characterization

## Abstract

Coatings have a fundamental role in covering the external surface of yachts by acting both as protective and aesthetic layers. In particular, fillers represent the essential layer from the point of view of mechanical properties and consist of a polymeric matrix, different extenders and additives, and dispersing agents, with the latter having the role to provide good extender-matrix compatibility. In the present work, the effects of dispersing agents with an ionic or steric action on the interactions between hollow glass microspheres and an epoxy-polyamide resin are evaluated. Un-crosslinked filler materials are studied via rheological tests, whereas the mechanical and morphological properties of the crosslinked samples are assessed. The results clearly indicate that steric dispersing agents provide a much greater compatibility effect compared to ionic ones, owing to their steric hindrance capability, thus leading to better-performing filler materials with a less-marked Payne effect, which is here proved to be an efficient tool to provide information concerning the extent of component interactions in nautical fillers. To the best of our knowledge, this work represents the first attempt to deeply understand the role of dispersing agents, which are until now empirically used in the preparation of fillers.

## 1. Introduction

Coating systems play a fundamental role in the construction of yachts and superyachts [1,2,3,4]. Indeed, by covering these external surfaces, coatings are most exposed to aggressive environments, such as seawater, marine atmosphere and thermal variations, thus providing a significant protection effect. Additionally, coatings must provide aesthetic properties typical of luxury products (e.g., brightness, light reflection, durability over time) [5,6,7,8]. Such performances are achieved through complex multilayer structures, namely painting systems. On the metallic substrate above the waterline, several layers with different thicknesses and functions are usually present. In particular, a bottom primer layer is covered by the filler and finishing filler strata, upon which another primer layer is applied before the undercoat and topcoat. The mechanical resistance of such a complex structure is mainly attributed to the filler/plaster layer, consisting in a composite material with a thickness of around 2 cm. Thus, it is not surprising that both the physicochemical features and the application of this layer are crucial for smoothing the surface, filling possible defects or voids and contributing to isolate the hulls [9]. However, few studies are available in the literature for these specific materials [5,6,10,11]. Fillers are usually made of two different parts consisting in an A component (e.g., epoxy resin) and a B component (e.g., curing agent based on a polyamide group), which, once mixed together in opportune ratios, form the final composite materials to be applied. In addition to the polymeric matrix, different types of additives, extenders and pigments are present in the formulation and require to be well dispersed in the matrix in order to exploit their functions [12,13]. The most common additives are rheological modifiers, antifoams and dispersing agents [5,12,14,15,16,17]. Rheological additives act on the viscosity of the samples, allowing to increase or reduce their tendency to flow and avoid the sagging phenomenon during the application step (i.e., thixotropic recovery), whereas anti-foams are used to obviate the formation of foams during the dispersion of extenders in the matrix phase [18,19,20]. The complexity of paint formulations is caused not only by their multicomponent composition but also by their multiphase nature and related thermodynamic instability [21,22,23,24]. In such a complex system, dispersing agents are essential to improve the incorporation of powders in the filler and ensure their stability during manufacturing, storage and application processes. The dispersing step is the most difficult and time/energy-consuming part of the entire paint manufacturing process, owing to the difference in surface tension between liquids (polymers and optional solvents) and powders (pigments and/or extenders) [25,26,27]. Dispersing agents are able to coat suspended powder particles to form a barrier that, either by ionic repulsion (i.e., an ionic dispersant generally having a low molecular weight) and/or steric hindrance (i.e., a non-ionic dispersant generally having a high molecular weight), prevents particle–particle interactions and aggregation. These agents, in comparison to the surfactants, are chemical compounds consisting in two well-defined parts: the oil soluble one (hydrophobic), with aliphatic or aromatic hydrocarbon residues, and the water soluble one (hydrophilic). The hydrophilic group can be ionic or non-ionic. In the first case, the stabilization mechanism is based on ionic repulsions with the formation of an electric layer (i.e., Helmholtz layer) on the particle surface, leading to electrostatic repulsive forces to guard against aggregation. Contrariwise, if the dispersing agents have a non-ionic nature, the stabilization mechanism is based on steric hindrance; these dispersants usually have pendant anchoring groups that are adsorbed onto the particle surface by hydrogen bonding, dipole–dipole interactions or Van der Waals forces. The free part of the chains is large enough to cause steric stabilization and to act as a bumper preventing the approach of particles to each other [28,29,30,31,32,33]. Concerning the extenders, whose function is mainly to reduce the density and cost of the fillers while maintaining satisfactory properties, the most commonly used are carbonates, talc, aluminosilicates and hollow glass microspheres [10,34,35,36,37].

The present work aims to deeply investigate the effect of different dispersing agents on the interactions between the polymer matrix and hollow glass microspheres in fillers for nautical applications. In particular, both ionic (i.e., based on soy lecithin and on diamine dioleate) and steric dispersants (i.e., based on hyperbranched polyester and phosphite titanate) are tested by evaluating the rheological, mechanical and morphological properties of the prepared fillers. To the best of our knowledge, this is the first time that the interactions between the extenders and the matrix are carefully investigated and, remarkably, the rheological results are discussed by taking into account the Payne effect and providing a new perspective in understanding the behavior of such products.

## 2. Materials and Methods

### 2.1. Materials

The formulations studied here were prepared ad hoc in order to underline the effect of different dispersing agents on the wettability of hollow glass microspheres. Tested samples consisted of an epoxy resin (component A), an anti-foam agent necessary to avoid foam formation, hollow glass microspheres used as extenders [38] and a proper dispersing agent. Rheological modifiers, solvents, other extenders and pigments were not used to simplify the studied formulations. Four samples, which were labeled from 1 to 4, were prepared by employing different dispersing agents. Sample 0 was without the dispersing agent and was employed as a reference. Table 1 summarizes the composition of the un-crosslinked samples. It should be noted that the additives used here were commercial products, and therefore, their specific compositions are not available; the nature of the selected additives is reported in Table 2.

In order to obtain crosslinked products for the mechanical and morphological investigations, a polyamide resin (B component) was added to each sample. The amount of B component was calculated as a function of the epoxy resin content in each sample (for samples 0 and 4, 40.5 and 40.6 *w/w*%, respectively; for samples 1 and 2, 3 and 40 *w/w*%, respectively).

Resins (i.e., A and B components), anti-foam agent, and hollow glass microspheres have been kindly provided by Boero Bartolomeo S.p.A. (Boero Bartolomeo S.p.A., Genova, Italy). Self-emulsifying soy lecithin has been supplied by Balestrini S.r.l (Balestrini S.r.l, Milan, Italy. N-tallow alkyl trimethylene diamine dioleate has been provided by Eurochemicals S.p.A. (Eurochemicals S.p.A., Cologno Monzese, Italy). Hyperbranched polyester has been supplied by BYK-Chemie GmbH (BYK-Chemie GmbH, Wesel, Germany). Tetra(2,2-diallyloxymethylene-1-butyl)bis(ditridecyl phosphite) titanate has been provided by Finco S.r.l (Finco S.r.l., Settimo Milanese, Italy).

### 2.2. Methods

The dispersion of microspheres and additives in the polymer matrix was carried out with a dissolver Dispermat LC30, 220V (Dispermat^®^, VMA-Getzmann GmbH, Columbia, MD, USA). The mixture temperature, speed and time during the dispersion were controlled in order to have a good dispersion. For instance, the temperature was maintained below 40 °C, and the dissolver speed was set at 150–200 rpm. For each sample, both the un-crosslinked and crosslinked products were studied.

The rheological measurements were performed on the un-crosslinked materials using an Anton Paar MCR 102 rheometer (Anton Paar GmbH, Graz, Austria), equipped with a 25-mm-diameter parallel plate geometry (PP25) and using a 1-mm gap. The rheometer was equipped with a Peltier heating system for the accurate control of the temperature. All measurements were set at 25.00 ± 0.01 °C. To evaluate the viscoelastic properties in terms of the storage modulus (i.e., G’, representing the storage and recovery energy in cyclic deformation), loss modulus (i.e., G”, representing the energy dissipated as heat) and complex modulus (i.e., G* = G”/G’), amplitude sweep tests (AS) with a deformation (γ) ranging from 0.02% up to 10% were performed at a fixed frequency of 1 Hz. The data were collected and analyzed using RheoCompass software (Anton Paar GmbH, Graz, Austria). Each sample was tested in triplicate to ensure result repeatability.

The mechanical and morphological characterizations were performed on the crosslinked products obtained by mixing the samples with a proper amount of polyamide. Three-point bending flexural tests were performed according to ASTM D790 standard through a dynamometer (Instron 3365, Norwood, MA, USA) at room temperature [39,40,41]. Measurements were performed in triplicate on the samples to ensure result repeatability. For the morphological characterization, a Zeiss Supra 40VP Scanning Electron Microscope (Carl Zeiss AG, Oberkochen, Germany) was used. The samples were thinly coated with gold and palladium (0.150 kÅ Au/Pd) using a physical vapor deposition instrument (Precision Etching Coating System, Model 682, Gatan Inc., Pleasanton, CA, USA) in order to obtain good conductivity. Manual image analysis was carried out on digitalized SEM images using the open-source ImageJ 1.51 software (National Institute of Health, Bethesda, MD, USA to measure the distance between the polymer matrix and the hollow glass microspheres.

## 3. Results

### 3.1. Rheological Measurements

**Figure 1:**
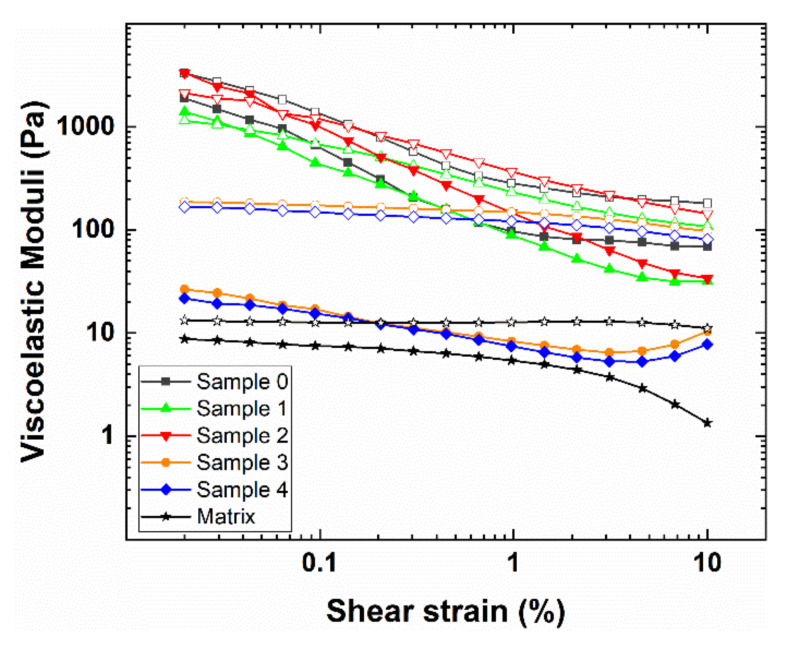
Dependence of the storage (filled symbols) and loss (empty symbols) moduli of Table 2. Dependence of the complex modulus of the tested samples upon the applied strain. The rheological response of the pure matrix is reported in the figure inset for comparison.

**Figure 2:**
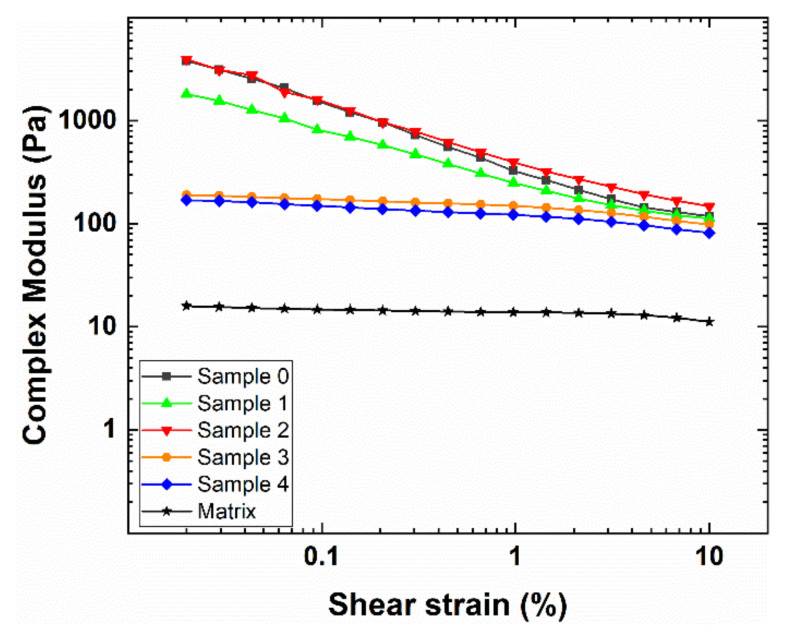
Dependence of the complex modulus of the tested samples upon the applied strain. The rheological response of the pure matrix is reported in the figure inset for comparison.

The rheological behavior of fillers is an important indicator of material applicability, as well as of the interactions occurring between their constituent components [18,42,43,44]. In particular, amplitude sweep tests are widely accepted to provide useful insights regarding the dispersion of extenders in a polymer matrix. Figure 1 and Figure 2 report the viscoelastic moduli (i.e., G’ and G”) and the complex modulus (i.e., G*) of the tested samples (i.e., 0–4), respectively, together with those of the simple matrix (i.e., pure epoxy resin without additives and microspheres).

First of all, except for samples 1 and 2 at really low γ values, the loss modulus G” always prevails over the elastic modulus G’ in the entire strain investigation range, therefore indicating that, in agreement with theory, the un-crosslinked materials show a prevalently viscous response [45]. Moreover, by comparing the matrix viscoelastic moduli with those of the samples (i.e., the polymer matrix with the added hollow glass microspheres), it can be noted that the presence of the extenders remarkably increases the material resistance, owing to their ability to both interact with the polymer matrix and form a secondary network [46]. Additionally, the tested samples can be clearly divided into two groups depending on their rheological behavior as a function of the applied strain. In more detail, samples 0, 1 and 2 present a high initial value of the moduli that rapidly decreases in around an order of magnitude with increasing γ. By contrast, samples 3 and 4 are characterized by lower initial values of G’, G” and G* that slowly, and only slightly, decrease at the larger strain. Such findings can be explained with the Payne effect, which is a typical response of rubber-based composites loaded with extenders [47,48,49]. Additionally, the mechanism responsible for the Payne effect is still controversial and not completely understood. The most commonly accepted explanation is related to the secondary network (extender–extender) formed by the extenders within the polymer matrix. At small amplitudes, this structure is able to act as a reinforcement, whereas it gets progressively destroyed upon the application of a greater oscillatory strain, leading to a marked decrease of the material resistance to solicitations. The larger the Payne effect, the greater the extender-extender interactions are at the expenses of those between the extenders and the matrix [50,51,52,53]. The marked Payne effect shown by samples 1 and 2, similar to that observed for sample 0 (i.e., the reference sample without a dispersing agent), is indicative of the fact that hollow glass microspheres are not efficiently dispersed and can form agglomerates, thus indicating the low efficiency of the ionic dispersing agents. On the other hand, the small Payne effect depicted for samples 3 and 4 clearly suggests the capability and proficiency of the steric dispersing agents in homogeneously dispersing the microspheres used, therefore promoting the extender-matrix interactions. To quantitatively evaluate the Payne effect of the tested samples, a widely employed approach consists in considering the Payne amplitude, ΔG*, as the difference between the complex modulus, G_0_*, at very low strain values (i.e., 0.02%), and the complex modulus, G_∞_*, at high strain values (i.e., 10%). The obtained results are summarized in Table 3.

In agreement with the rheological response reported in Figure 1 and Figure 2, and taking into account the above discussion, the Payne amplitude evaluation clearly demonstrates that additives 3 and 4 perform well in dispersing hollow glass microspheres within the polymer matrix and are able to provide a response similar to that of the polymer matrix. Conversely, additives 1 and 2 offer a negligible, or even negative, dispersing effect and do not provide any significant difference compared to sample 0 (i.e., the reference sample). Owing to the different nature of the dispersing agents used, such findings provide the first evidence that steric surfactants are much more efficient in the investigated fillers by exploiting their dispersing action due to steric hindrance [54,55].

### 3.2. Mechanical Tests

Compared to rheological tests performed on un-crosslinked formulations, the mechanical response of solid crosslinked materials can be employed to evaluate the reinforcing effect of extenders in nautical fillers. Indeed, the predominance of extender-extender interactions results in poor mechanical performance due to the impossibility to efficiently transfer an applied stress between the material components with the consequent formation of weak spots; conversely, a good compatibility between the extenders and the matrix allows obtaining a much more homogeneous and performing material with an enhanced response compared to the pure polymer [56]. Here, mechanical bending tests were performed on samples with a thickness of 0.8 cm, a width of 2.0 cm and a length of 20 cm. In particular, the Young modulus (E_b_), the bending strength (σ_b_) and the deformation at break (ε_b_) were calculated, with the results summarized in Table 4.

In agreement with the rheological results, two sample groups can be clearly individuated on the basis of their mechanical response. In detail, using ionic dispersants (i.e., samples 1 and 2) was found to decrease both the break strength and elongation of the system with values of around 18 MPa and 1.3%, respectively, and no differences were observed for the elastic modulus (i.e., ~1500 MPa) with respect to sample 0. The observed break strength decrement is probably ascribable to a slight plasticizing action caused by the ionic dispersants [33,57,58], also taking into account that the studied hollow glass microspheres offer a modest reinforcement effect compared to other filler types. By contrast, the steric dispersants (i.e., samples 3 and 4) induced a considerable increment of the system elastic modulus, without affecting the break strength and only slightly reducing the filler deformability [59,60,61]. Compared to the rheological results, such findings indicate that steric dispersants are able to provide a much more marked compatibilization effect for the studied system (i.e., hollow glass microspheres embedded in an epoxy resin matrix), as well as the fact that ionic dispersants are almost completely ineffective and can even to some extent worsen the filler mechanical response.

### 3.3. Morphological Characterization

**Figure 3:**
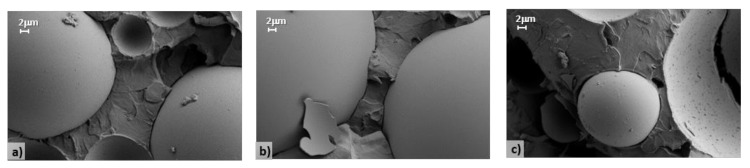
SEM images of samples (**a**) 0, (**b**) 1, and (**c**) 2.

**Figure 4:**
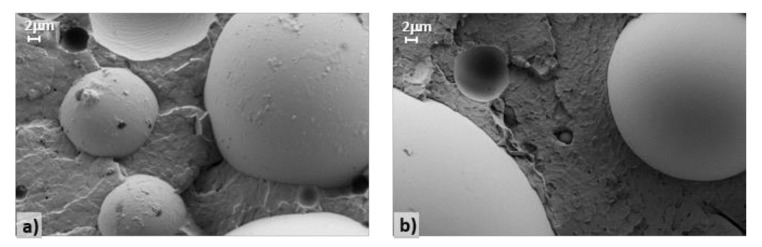
SEM images of samples (**a**) 3 and (**b**) 4.

A simple and fast approach to qualitatively estimate extender-matrix interactions in crosslinked fillers relies on evaluating the distance between the two components via morphological characterization. SEM images for samples 0, 1 and 2 and for samples 3 and 4 are reported in Figure 3 and Figure 4, respectively, with the extender-matrix distance (d) summarized in Table 4.

As clearly shown, the glass microsphere wettability strongly depends on the employed dispersing agents and reflects the rheological and mechanical results. Sample 0 (Figure 3a) is characterized by a well-defined empty region between the extenders and the polymers (d = 0.254 μm), thus suggesting the complete incompatibility between these components. Similarly, samples 1 (Figure 3b) and 2 (Figure 3c) present the same morphology of the reference sample, in addition to the presence of the dispersing agents (d = 0.235 and 0.249 μm, respectively), which consequently can be considered totally unable to create effective interactions between the extenders and the matrix. Conversely, samples 3 (Figure 4a) and 4 (Figure 4b) are characterized by a different morphology, where a neat interface between the components cannot be clearly depicted, thus proving their good compatibility and the existence of a continuous composite structure with enhanced performance. Note that it was not possible to calculate the extender-matrix distance for the last two samples.

**Figure 5:**
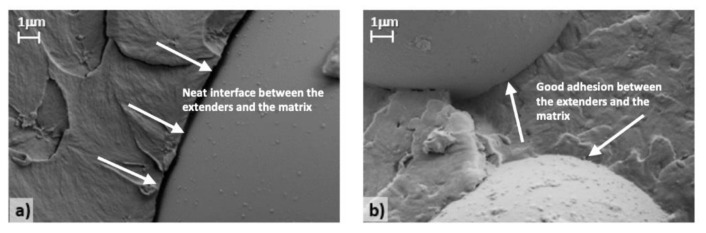
High-magnification SEM images of samples (**a**) 2 and (**b**) 3.

To better visualize the described phenomenon, Figure 5 shows the SEM images at high magnification of sample 2 (Figure 5a), which is characterized by the presence of an ineffective dispersing agent, and sample 3 (Figure 5b), which is instead characterized by the presence of an efficient dispersing agent. As clearly highlighted by the white arrows, a neat extender-matrix interface can be observed in Figure 5a. By contrast, an almost continuum medium with a slightly detectable interface is depicted in Figure 5b.

## 4. Conclusions

In the present work, the effect of dispersing agents with a different action mode (i.e., ionic or steric) on the interaction between hollow glass microspheres, which were used as extenders, and an epoxy-polyamide resin, which represented the typical polymer matrix used in nautical fillers, was investigated. The rheological behavior of the studied samples clearly indicated that the performance of the steric surfactants was much more enhanced in reducing the extender-extender interactions, compared to the ionic ones. In more detail, the Payne effect, which consists of a marked decrease of the material viscoelastic moduli G’ and G” upon the application of an oscillatory shear, was found to be much more evident in the presence of the ionic additives, thus indicating their poor efficiency in homogeneously dispersing the microspheres. Additionally, bending tests proved that the steric dispersants improved the mechanical resistance of the fillers, owing to their capability to form a continuous complex structure with an enhanced response. Remarkably, the sample morphological investigation allowed for the clear visualization of the effect of the different dispersing agents on the wettability of the glass extenders; in particular, whereas a neat interfacial region could be detected for the ionic surfactants, the steric ones led to a much greater adhesion of the two components, reflecting the previous findings. However, further experiments are clearly needed to fully understand the described phenomenon. This work represents the first scientific report concerning the evaluation of the effect of different dispersing agents on the performance of fillers in the nautical field.

## Figures and Tables

**Table 1:** Summary of the un-crosslinked sample compositions expressed in *w/w*%.

Label	Component A	Dispersing Agent	Microspheres	Anti-Foam Agent
Sample 0 (without dispersing agent)	81.2	0.0	18.3	0.5
Sample 1 (with additive 1)	80.0	1.5	18.0	0.5
Sample 2 (with additive 2)	80.0	1.5	18.0	0.5
Sample 3 (with additive 3)	80.0	1.5	18.0	0.5
Sample 4 (with additive 4)	81.0	0.1	18.4	0.5

**Table 2:** Chemical nature of the dispersing agents used.

Dispersing Agent	Nature	Label
Additive 1	Ionic dispersants	Self-emulsifying soy lecithin
Additive 2		N-tallow alkyl trimethylene diamine dioleate
Additive 3	Steric dispersants	Hyperbranched polyester
Additive 4		Tetra(2,2-diallyloxymethylene-1-butyl)bis(ditridecyl phosphite) titanate

**Table 3:** The complex moduli, G_0_* (at very low strain) and G_ꝏ_* (at high strain), and Payne amplitude (ΔG*) values for the tested polymer matrix and samples.

Sample.	G_0_* (Pa)	G_∞_* (Pa)	ΔG* (Pa)
Matrix	15 ± 1	11 ± 0.5	4.8 ± 0.9
Sample 0	3812 ± 11	119 ± 1	3693 ± 11
Sample 1	1733 ± 70	104 ± 9	1629 ± 61
Sample 2	3951 ± 4	148 ± 1	3803 ± 4
Sample 3	193 ± 4	101 ± 3	91 ± 1
Sample 4	169 ± 2	83 ± 4	87 ± 3

**Table 4:** Summary of the samples’ mechanical and morphological properties.

Sample	E_b_ (MPa)	σ_b_ (MPa)	ε_b_ (%)	Extender–Matrix Distance (μm)
Sample 0	1454 ± 90	24.9 ± 1.8	1.80 ± 0.17	0.254 ± 0.047
Sample 1	1444 ± 31	17.7 ± 0.9	1.34 ± 0.11	0.235 ± 0.023
Sample 2	1490 ± 53	18.0 ± 0.7	1.26 ± 0.07	0.249 ± 0.051
Sample 3	1866 ± 32	24.9 ± 1.3	1.38 ± 0.13	-
Sample 4	1857 ± 59	24.4 ± 1.5	1.36 ± 0.14	-
